# Biological research on the occurrence and development of multiple myeloma and its treatment

**DOI:** 10.1002/iid3.850

**Published:** 2023-05-08

**Authors:** Yue Wu, Xiangjun Shi, Xinchen Yao, Xinru Du

**Affiliations:** ^1^ Department of Orthopaedics Beijing Chao‐Yang Hospital Beijing China

**Keywords:** biological research, hematopoietic stem cell transplantation, immunomodulatory drugs, immunotherapy, multiple myeloma, proteasome inhibitor

## Abstract

**Introduction:**

To review the pathogenesis and treatment of multiple myeloma (MM). MM is a hematological malignancy with abnormal plasma cell proliferation in bone marrow. Due to the emergence of drug resistance, MM is still an incurable malignancy, which requires further exploration of pathogenesis and effective therapeutic targets.

**Methods:**

In this paper, the method of literature review is adopted to obtain the information about MM. Based on the literature, comprehensive and systematic review is made.

**Results:**

MM is a complex pathophysiological process with great heterogeneity, mainly reflected in genomic instability and bone marrow microenvironment. At present, the treatment of MM has made great progress, proteasome inhibitors and immunomodulatory drugs are widely used in clinic. Allogeneic stem cell transplantation may be the only promising cure for MM, and its high transplant‐related mortality limits its clinical application.

**Conclusions:**

The future of MM treatment lies in the development of more targeted therapies, novel immunotherapies, and a better understanding of the disease's molecular and genetic basis.

## Graphical abstract

Although the proteasome inhibitors and immune modulators scheme improved the overall prognosis of patients, cellular immune therapy and hematopoietic stem cell transplantation for the treatment of multiple myeloma also opens up a wider prospect, but disease recurrence and resistance and treatment‐related toxicity is still the important factors that affect the patient's prognosis and survival, seek a more safe and effective treatment strategies is particularly important.

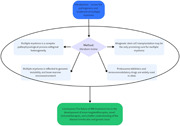

## 1 INTRODUCTION

Multiple myeloma (MM) is a hematological cancer that originates from plasma cells, a type of white blood cell found in the bone marrow. Plasma cells are essential for producing antibodies that help the body fight infections and maintain a healthy immune system. In MM, abnormal plasma cells multiply uncontrollably, forming tumors within the bone marrow and crowding out healthy blood cells. This leads to various health complications, such as anemia, bone pain, kidney damage, and a weakened immune system, making patients more susceptible to infections.^1^, ^2^ This disease occurs between 50 and 60 years old, and is a common malignant disease in the department of hematology.^3^ About 80% patients are complicated with osteolytic lesions, namely MM bone disease.^4^

At the genetic level, MM often presented with chromosome number or structure abnormalities, such as oncogene and immunoglobulin heavy chain (IGH) region translocation, hyperdiploid, and so forth.^5^, ^6^, ^7^ In addition, a large number of gene mutations were found in whole genome and exon sequencing, which may be involved in the pathological changes of diseases. The treatment of MM has improved significantly over the past 20 years as the biological understanding of MM has increased, and as more and more new drugs were approved for the treatment of relapse‐resistant MM, the treatment of MM is gradually moving from a nonspecific era to a new era of targeted therapy.^8^, ^9^ In this study, the pathogenesis and treatment of MM are reviewed, and the aim of this study is to make the clinical medical workers better study the pathogenesis and treatment of MM.

## 2 THE PATHOGENESIS OF MM

### 2.1 Genomic instability

The pathogenesis of MM is a multigene, multi‐stage and multi‐step process, and almost all patients experience a relatively benign process called monoclonal gammopathy of undetermined significance to smoldering multiple myeloma and then to active multiple myeloma, in which genomic instability leads to the accumulation of genetic abnormal events and partial chromosomal changes.^10^, ^11^ Cytogenetic abnormality is one of the main basis for risk stratification of MM. The patients can be roughly divided into two categories according to chromosomal structure and numerical abnormalities: hyperdiploid and non‐hyperdiploid.^12^ Odd chromosome trisomy usually occurs in hyperdiploids, mainly including +3, +5, +7, +9, +11, +15, +19, and +21.^13^ Non‐hyperdiploid genes are often associated with translocations between IgH genes and other chromosomes.^14^ Chromosomal abnormalities often lead to increased expression levels of multiple cell cycle related genes, such as CCND1, CCND2, CCND3, and so forth, and promote the continuous proliferation of long‐lived plasmacell, which is one of the important reasons for the occurrence of MM.^15^, ^16^

### 2.2 Bone marrow microenvironment

Bone marrow microenvironment contains complex components, which can be composed of bone marrow stromal cells (BMSCs), osteoclasts (OCs), osteoblasts, mesenchemal stem cells (MSCs), macrophages, immune cells and other cellular components, as well as cytokines, chemokines, growth factors, extracellular matrix and other noncellular components.^17^, ^18^, ^19^ The direct contact between MM cells and nontumor cells in bone marrow microenvironment can secrete soluble cytokines for signal transmission so that tumor cells can obtain growth signals, which is conducive to the formation of immunosuppressive tumor microenvironment.^20^, ^21^ In addition, abnormal immune cell function in microenvironment also plays a key role in the pathogenesis of MM.

Exosomes are small vesicles released into the extracellular environment by various types of cells under physiological or pathological conditions, which can deliver mRNAs, miRNAs and proteins through the fusion of cell membranes or endocytosis of recipient cells to regulate the biological behavior of cells.^22^ Exosomes derived from MM cells can increase the phosphorylation level of Signal transducers and activators of transcription 3 (STAT3) and up‐regulate antiapoptotic protein B‐cell lymphoma.^23^ The expression of BCL‐XL and myeloid cell leukemia I (MCL‐ I) can activate myeloid‐derived suppressor cells (MDSC), enhance its immunosuppressive ability to T cells, and promote the growth of MM.^24^ MM cell‐derived exosomes are rich in a variety of angiogenesis related proteins, such as osteopontin, CXC chemokineligand 16 (CXCL16), endothelin‐1, etc., which promote endothelial cell growth and angiogenesis and have been proven to be one of the factors contributing to poor prognosis of patients.^25^, ^26^, ^27^ In addition, MM cell‐derived exosomes have been shown to accelerate osteolysis and promote MM formation.^28^ By activating the serine/threoninekinase (AKT) signaling pathway and upregulating the expression of antiapoptotic protein, exosomes derived from MM cells inhibit the apoptosis of osteoclast precursor cells, promote the survival and migration of OCs, and induce their differentiation into OCs, thus promoting the bone resorption activity of OCs.^29^ After the increase of the number and activity of OCs, the proliferation and survival of MM cells were further promoted by the secretion of IL‐6 and B‐cell activating factors and the strengthening of their contact with MM cells.^30^, ^31^

## 3 DISEASE STAGING IN MM

Disease staging is crucial for determining the severity of MM, predicting patient outcomes, and guiding treatment decisions. Over the years, several staging systems have been developed to classify MM patients based on specific clinical and laboratory factors. Here, we discuss three prominent staging systems: the Durie‐Salmon system, the International Staging System (ISS), and the Revised ISS (R‐ISS).

### 3.1 Durie‐Salmon system

The Durie‐Salmon system, developed in the 1970s, classifies MM patients into three stages (I, II, and III) based on the levels of serum albumin, serum calcium, hemoglobin, and the extent of bone lesions. Each stage has two substages (A and B) depending on the kidney function. Although the Durie‐Salmon system has been widely used for many years, it has limitations, as it does not consider newer prognostic factors and may not accurately reflect the patient's prognosis.^32^, ^33^, ^34^, ^35^

### 3.2 ISS

Introduced in 2005, the ISS is a simpler staging system that uses only serum beta‐2 microglobulin and albumin levels to classify patients into three stages (I, II, and III). The ISS system has been shown to better predict the survival outcomes of MM patients compared to the Durie‐Salmon system. However, the ISS system does not take into account genetic abnormalities, which are known to impact prognosis significantly.^36^, ^37^, ^38^

### 3.3 R‐ISS

To further refine the prognostic accuracy, the R‐ISS was developed in 2015. It combines the ISS system with additional factors, such as lactate dehydrogenase levels and high‐risk cytogenetic abnormalities, to classify patients into three stages (R‐ISS I, R‐ISS II, and R‐ISS III). The R‐ISS has been demonstrated to provide a more accurate prognosis for MM patients, allowing clinicians to select appropriate treatment strategies based on the patient's stage.^39^, ^40^, ^41^

## 4 THE TREATMENT OF MM

The incidence of MM ranked second among hematological malignancies, accounting for about 10%.^42^ The new cases and mortality rate from 2011 to 2016 were 6. Nine cases per 100,000 population and 3.3 cases per 100,000 people, with an increasing trend year by year, especially among the middle‐aged and elderly.^43^ Its overall survival is not optimistic, the 5‐year survival rate of newly diagnosed patients is about 47·3%−53·8%.^44^ The treatment of MM has improved significantly over the past 20 years, with patients continuing to benefit from effective treatments with acceptable toxicity, such as the use of proteasome inhibitors. As more and more new drugs are approved or being developed for the treatment of relapsing and refractory MM, the diagnosis and treatment of MM is gradually moving from a nonspecific era to a new era of genetic testing and targeted therapy.

### 4.1 Proteasome inhibitor

Preclinical studies have shown that proteasome inhibitors can cause intracellular protein accumulation in malignant plasma, leading to activation of unfolded protein stress response and reduction of NF‐B activity, both of which induce apoptosis.^45^, ^46^ Three proteasome inhibitors are currently approved for the treatment of patients with MM: bortezomib, carfilzomib and ixasomib. Bortezomib is a first‐generation reversible proteasome inhibitor, which can specifically inhibit the activity of proteasome, inhibit the expression of genes related to cell proliferation, reduce the secretion of myeloma cell growth factors such as IL‐6 and the expression of adhesion factors, and ultimately lead to tumor cell apoptosis.^47^ Carfilzomib is a tetrapeptide epoxy ketone that selectively targets and irreversibly inhibits the proteasome. Compared with bortezomib, carfilzomib has higher selectivity for proteasome β5 subunit and lower untargeted activity against nonproteasome proteases, showing significant efficacy and low toxicity in relapsed/refractory MM.^48^ Ixasomib is a reversible proteasome inhibitor that preferentially binds to the β5 subunit of the 20 S proteasome and inhibits its activity.^49^ In November 2015, the FDA approved ixasomib in combination with linalidomide and dexamethasone for the treatment of MM in patients receiving at least one therapy.^50^ Ixasomib is currently the only approved oral three‐drug regimen for relapsed/refractory MM. Preclinical studies have shown that ixasomib exhibits more favorable pharmacokinetic, pharmacodynamic and antitumor activity than bortezomib.^51^

### 4.2 Immunomodulatory drugs

In recent years, the application of immunomodulatory drugs represented by thalidomide and its derivative lenalidomide not only improves the remission rate and the remission depth of MM patients, but also significantly prolongs the survival time of patients. Drug lenalidomide is a second generation immunomodulatory drug, can be produced by triggering T cells induced IL‐2, NK cell‐mediated cytotoxicity and antibody dependent cytotoxicity, play a role of immune regulation, and also can inhibit angiogenesis, inhibiting cytokines and BMSCs mediated tumor cells resistant to produce, myeloma cell apoptosis.^52^, ^53^ Compared with thalidomide, its immunomodulatory and antitumor effects are stronger, and its toxicity is lower than that of thalidomide.

Pomalidomide‐third generation immunomodulatory drug, like other immunomodulatory drugs, acts through the CRBN gene and binds to CRBN to form the CRBN‐Cul4A‐DDB1‐Roc1 complex, which has E3 ubiquity ligase activity.^54^ The tumor cell transcription factors IKZF1 and IKZF3 were ubiquitinated, after which the ubiquitinated IKZF was degraded by the proteasome, thus stopping the growth of myeloma cells.^55^ PD regimen of pomalidomide + dexamethasone is mainly used in RRMM patients who are resistant to thalidomide and lenalidomide. The results showed that PFS in the pemadomide + low‐dose dexamethasone group was significantly longer than that in the high‐dose dexamethasone group (4.0 v. 1.9 months, *p* < .0001), OS was significantly improved (12.7 v. 8.1 month).^56^ In addition, permadomide is also effective in high‐risk cytogenetic patients who containing 17 P deletion and (4; 14) translocation genes.^57^

### 4.3 Immunotherapy

In recent years, tumor immunotherapy has shown great potential for a variety of malignancies, including MM. The human immune system is diverse and specific, relying on a variety of effector mechanisms, such as Fas ligand, complement system, perforin, granular enzyme and interferon γ.^58^, ^59^, ^60^, ^61^ Immunotherapy includes chimeric antigen receptor T cell immunotherapy, monoclonal antibody, bispecific T cell conjugation antibody (BiTEs), bispecific antibody, immune checkpoint inhibitor, and so forth.

CAR T is produced from autologous T cells using lentivirus or retrovirus vector‐mediated transfection technology, which can recognize specific antigens expressed on tumor cells and then kill tumor cells.^62^ CAR T cells targeting CD19 are the most successful and widely studied to date. CD19 is a surface protein expressed in precursor and mature B cells and is a target of diseases such as acute B‐cell lymphoma and non‐Hodgkin's lymphoma.^63^, ^64^ CD19 is usually not expressed on the surface of cloned plasma cells. Because B cell Maturation antigen (BCMA) is highly expressed on the surface of cloned plasma cells, it is an ideal target for CAR T cells in MM.^65^ In the treatment of MM patients with CAR‐T, many problems need to be solved, such as the selection of target antigen, the control of cytokine release syndrome and other adverse reactions, and the reduction of huge cost. As a result, novel CAR‐T cell therapies are still under development.

### 4.4 Hematopoietic stem cell transplantation (HSCT)

HSCT is a mature technique for the treatment of hematologic diseases. Initial treatment for MM includes autogenous HSCT (AUTO‐ASCT) and allogeneic HSCT (ALLO‐SCT).^66^, ^67^ Allogeneic stem cell transplantation may be the only promising cure for MM. As early as 2011, a study in EBMT evaluated the efficacy of sequential auto‐AlloHSCT and single ASCT in MM patients, and PFS of sequential Auto‐AlloHSCT was superior to single ASCT at 60 months of follow‐up (33% vs. 18%), and there was no significant difference in OS rate (64% vs. 57%), sequential Auto‐AlloHSCT OS was significantly better than single ASCT at 96 months of follow‐up (49% vs. 36%).^68^ Although ALLO‐SCT can improve patients' PFS, there are still many problems to be solved in this field. ALLO‐SCT is prone to graft versus host disease and has a high transplant‐related mortality.^69^ Early infection after transplantation has become one of the main causes of death in HSCT patients. Infection can be manifested in oral mucosa, upper respiratory tract, lung, perianal, blood, venous catheter, urinary tract, intestinal tract and skin and soft tissue, often accompanied by co‐infection in different parts.^70^, ^71^ High‐dose Mel (200 mg/m^2^) pretreatment plus autologous HSCT (ASCT) is the standard treatment for young patients with MM.^72^ Several studies over the years have shown that this regimen improves progression‐free survival in these patients, and early transplantation significantly improves progression‐free survival.

### 4.5 Monoclonal antibodies (mAbs)

mAbs are laboratory‐produced molecules designed to target specific antigens present on the surface of cancer cells or other harmful agents. These antibodies can bind to specific proteins or other molecules on the surface of target cells, leading to various outcomes such as neutralization, immune system activation, or cell death.^73^, ^74^ In MM, several mAbs have been developed to target specific antigens on the surface of malignant plasma cells, resulting in improved treatment outcomes and prolonged survival for patients.

Daratumumab is a monoclonal antibody that targets CD38, a protein highly expressed on the surface of MM cells. By binding to CD38, daratumumab triggers the immune system to attack and eliminate myeloma cells.^75^, ^76^ It has been approved for use as a single agent and in combination with other therapies, such as lenalidomide, bortezomib, or pomalidomide, for the treatment of MM in various stages, including newly diagnosed, relapsed, and refractory disease.^77^, ^78^, ^79^, ^80^ Daratumumab has shown significant efficacy in improving progression‐free survival and overall survival in MM patients.

Elotuzumab is a monoclonal antibody that targets SLAMF7 (Signaling Lymphocytic Activation Molecule Family member 7), a protein expressed on the surface of myeloma cells and natural killer (NK) cells.^81^ Elotuzumab enhances the activity of NK cells, promoting the immune system's ability to target and destroy myeloma cells.^82^, ^83^ It is approved for use in combination with lenalidomide and dexamethasone or with pomalidomide and dexamethasone for the treatment of relapsed or refractory MM.^84^, ^85^, ^86^ Clinical trials have demonstrated that elotuzumab, when combined with these therapies, significantly improves progression‐free survival in patients with relapsed or refractory disease.

Isatuximab is another monoclonal antibody targeting CD38, similar to daratumumab. It binds to a specific region of CD38, leading to the destruction of myeloma cells through several mechanisms, including antibody‐dependent cellular cytotoxicity, complement‐dependent cytotoxicity, and immune‐mediated phagocytosis.^87^, ^88^ Isatuximab has been approved in combination with pomalidomide and dexamethasone for the treatment of relapsed or refractory MM. Clinical studies have shown that the addition of isatuximab to pomalidomide and dexamethasone significantly improves progression‐free survival in patients with relapsed or refractory disease^89^, ^90^, ^91^ (Table 1).

**Table 1:** The drugs of MM.

Treatment category	Specific treatments/drugs	Description/function
Proteasome Inhibitors	Bortezomib, Carfilzomib, Ixazomib	Induce apoptosis by causing intracellular protein accumulation and reducing NF‐κB activity; approved for use in MM patients
Immunomodulatory drugs	Thalidomide, Lenalidomide, Pomalidomide	Improve remission rates and survival time for MM patients by modulating immune response, inhibiting angiogenesis, and inducing myeloma cell apoptosis
Immunotherapy	CAR‐T, BiTEs, bispecific antibodies, immune checkpoint inhibitors	Engage the immune system to recognize and eliminate myeloma cells; new and developing treatment strategies
Monoclonal Antibodies	Examples: Daratumumab, Elotuzumab	Target specific antigens on myeloma cells and recruit the immune system to eliminate them; approved for use in MM patients, often in combination with other therapies
Hematopoietic Stem Cell Transplantation (HSCT)	Autologous (AUTO‐ASCT), Allogeneic (ALLO‐SCT)	Mature technique for MM treatment, with AUTO‐ASCT as the standard treatment for eligible patients; improves progression‐free survival and overall survival

Abbreviation: MM, multiple myeloma.

## 5 CONCLUSIONS

MM is a type of malignant tumor occurring in incompletely differentiated plasma cells. Genomic instability and bone marrow microenvironment maybe the main pathogenesis of MM. Although the proteasome inhibitors and immunomodulatory drugs scheme improved the overall prognosis of patients, cellular immune therapy, mAbs and HSCT for the treatment of MM also opens up a wider prospect, but disease recurrence and resistance and treatment‐related toxicity is still the important factors that affect the patient's prognosis and survival, seek a more safe and effective treatment strategies is particularly important.

## AUTHOR CONTRIBUTIONS

Xiangjun Shi and Xinchen Yao contributed to the data collection and performed the data analysis. Yue Wu wrote the manuscript. Xinru Du contributed to the critical revision of article. All authors read and approved the final manuscript.

## CONFLICT OF INTEREST STATEMENT

The authors declare no conflict of interest.

## Data Availability

The data supporting this review are from previously reported studies and datasets, which have been cited. The processed data are available from the corresponding author upon request.
